# Targeted adjustment of the posterior tibial slope in unicompartmental knee arthroplasty is feasible without altering the medial proximal tibial angle

**DOI:** 10.1002/jeo2.70286

**Published:** 2025-05-19

**Authors:** Moses K. D. El Kayali, Rosa Berndt, Clemens Gwinner, Lorenz Pichler

**Affiliations:** ^1^ Center for Musculoskeletal Surgery Charité – Universitätsmedizin Berlin Berlin Germany; ^2^ Department of Orthopedics and Trauma‐Surgery Medical University of Vienna Vienna Austria

**Keywords:** alignment, knee osteoarthritis, MPTA, posterior tibial slope, unicompartmental knee arthroplasty

## Abstract

**Purpose:**

Recommendations regarding the surgical modification of the medial posterior tibial slope (mPTS) in medial unicompartmental knee arthroplasty (UKA) vary. Given the high preoperative variability, this often results in a significant change in the patient's mPTS through UKA. However, it is unclear whether this change in mPTS impacts the coronal alignment, specifically the medial proximal tibial angle (MPTA). Therefore, the purpose of this study was to report on the preoperative to post‐operative changes in mPTS and MPTA and their potential correlation in UKA.

**Methods:**

Pre‐ and post‐operative radiographs of 96 consecutive patients undergoing conventional medial UKA were analyzed. Pre‐ and post‐operative mPTS and MPTA were measured on radiographs by two observers and reported. Their differences, as well as the difference from the target value of 7°, were analyzed. Cases were grouped regarding their mPTS change into cases with <3° and cases with ≥3° mPTS change, and the correlation between changes in mPTS and changes in MPTA was reported.

**Results:**

The mean mPTS was reported at 9.27° (standard deviation [SD], 3.41°) preoperatively and 7.25° (SD, 2.23°) post‐operatively, with a mean change of −2.02° (SD, 3.84°; *p* < 0.001). Overall, 71.7% of cases had a post‐operative mPTS within ±2° of 7° without significant difference from the target value of 7° (*p* = 0.797). At a mean preoperative MPTA of 85.39° (SD, 2.34°) and a mean post‐operative MPTA of 84.12° (SD, 2.55°), UKA resulted in an average change of MPTA of −1.28° (SD, 2.55°; *p* < 0.001). Correlation coefficients revealed very weak correlations between the change in mPTS and the change in MPTA for all groups (*r* < −0.13 in all).

**Conclusions:**

Targeted mPTS modification can be achieved, which implies a significant change from preoperative mPTS values in patients undergoing UKA. However, the change in mPTS does not affect the change in MPTA.

**Level of Evidence:**

Level III.

**Abbreviations**
ACL: anterior cruciate ligament
ANOVA: analysis of variance
BMI: body mass index
MPTA: medial proximal tibial angle
mPTS: medial posterior tibial slope
PTS: posterior tibial slope
SD: standard deviation
TKA: total knee arthroplasty
UKA: unicompartmental knee arthroplasty

## INTRODUCTION

Unicompartmental knee arthroplasty (UKA) is an effective treatment for patients with medial compartment disease of the knee and has seen increased use in recent years [15]. Despite its success, data from national registries indicate an underuse of UKA for treating medial unicompartmental osteoarthritis [2, 4, 23]. Concerns regarding higher revision rates compared to total knee arthroplasty (TKA) appear to be a contributing factor [6, 12, 20].

Higher revision rates can result from unfavourable component positioning and lower limb malalignment, leading to early polyethylene wear, progression of lateral compartment osteoarthritis and aseptic loosening of implant components [21, 36]. In the coronal plane, overcorrection to valgus can result in contralateral compartment degeneration and early loosening, which should be avoided [10, 11, 16, 35].

However, there is limited evidence on sagittal alignment in UKA, specifically regarding the medial posterior tibial slope (mPTS), with manufacturer recommendations for optimal mPTS varying [38]. Furthermore, it is unclear whether target mPTS values can be consistently achieved with conventional, non‐robotic UKA.

Modifications in the sagittal plane have been observed to unintentionally affect coronal alignment. This relationship has been well‐documented in high tibial osteotomies, where changes in the posterior tibial slope (PTS) can lead to unintended changes in the medial proximal tibial angle (MPTA) [3, 5, 25]. However, it remains unknown whether a similar interaction occurs during UKA. A possible explanation for this interaction is the occurrence of bone‐cutting errors during tibial resection, which have been reported to contribute to malalignment in both TKA and UKA [14, 18, 30, 41]. Studies indicate that these errors are more frequent in the sagittal than in the coronal plane, particularly in conventional (non‐robotic) UKA, where the absence of navigation systems prevents real‐time monitoring and correction of cutting deviations [14].

This effect may be particularly relevant in cases with a preoperative mPTS that deviates significantly from the target range, requiring substantial intraoperative mPTS modification. In such cases, extensive sagittal plane modifications could inadvertently alter coronal alignment, affecting implant positioning and overall limb alignment.

Given that most partial knee systems specify a target range for the MPTA, it is crucial to determine whether and to what extent mPTS modifications influence coronal alignment.

Therefore, this study reports on the mean changes in mPTS and MPTA among patients undergoing conventional, non‐robotic‐assisted UKA and their correlation. Our first hypothesis is that an accurate modification of the mPTS within ±2° of the manufacturer's recommendation can be achieved in the majority of patients. Our second hypothesis is that the MPTA target range can be met even in patients with abnormal preoperative mPTS values necessitating ≥3° of mPTS change.

## MATERIALS AND METHODS

### Patients

A total of 410 patients who underwent conventional unilateral medial UKA at a high‐volume academic orthopaedic surgery centre by a single, highly experienced surgeon between March 2015 and May 2023 were screened for inclusion. Inclusion criteria followed the recommendations of the manufacturer of the knee system used in all patients (Oxford Partial Knee System) [28] and were defined as follows: primary, unilateral medial UKA performed on either the left or right knee, availability of pre‐ and post‐operative knee radiographs, completeness of patient records, and patient consent.

Exclusion criteria were defined as follows: knee radiographs not meeting quality criteria as delineated under ‘Radiographs’, insufficient patient records, previous ligamentous surgery or osteotomies, absence of a radiographic reference ball, UKA performed by other surgeons in our centre and the use of other unicompartmental knee systems.

Data collected included demographic information (age, gender and body mass index), surgical data (date of surgery and implant design), as well as pre‐ and post‐operative knee radiographs. Demographic data are presented in Table 1.

**Table 1:** Patient demographics.

	Male	Female	Overall
*n*	45 (46.87%)	51 (53.13%)	96 (100%)
Age, years	69.71 (range: 51.27–85.13)	66.61 (range: 46.31–82.82)	68.26 (range: 46.23–85.44)
BMI	28.89 (range: 22.70–43.20)	27.95 (range: 20.70–37.60)	28.43 (range: 20.70–43.20)

*Note*: Values presented represent means if not stated otherwise.

Abbreviation: BMI, body mass index.

### Surgical technique

All cases were performed using the Microplasty Instrumentation for the Oxford Partial Knee System (Zimmer Biomet) and in accordance with the surgical technique guidelines provided by the manufacturer. A standard parapatellar approach to the knee, a tourniquet, a mobile bearing insert and fixation by cementation were applied in all cases. According to the surgical technique guidelines of the manufacturer, a post‐operative mPTS of 7° was aimed for, using the Microplasty tibial saw guide. With the goal of slight remaining varus, the target range for the MPTA was set at 87 ± 5° [32, 34].

### Radiographs

Lateral, short, weightbearing and anterior‐to‐posterior long leg weightbearing radiographs of the respective knee were obtained at the time of indication for UKA as well as post‐operatively. All post‐operative radiographs were obtained during the early post‐operative period, before inpatient hospital discharge, within 10 days after surgery.

Lateral radiographs were standardized by positioning the knee joint 120° flexion, with the detector aligned parallel to the sagittal plane, and the central focus directed at the patellofemoral joint line. For long leg radiographs, standardization included fully extending the knees, placing the feet 10 cm apart with a 10° external rotation using a positioning template, positioning the hands at the sides and ensuring equal weight distribution across both legs.

Both pre‐ and post‐operative radiographs were calibrated using a standard reference ball with a diameter of 25.4 mm (1 in.).

Radiographs were taken using a digital radiography system (XGEO GC85A, Samsung). For long leg radiographs, three separate images were obtained and stitched via software (S‐Station, Version 3.05, Samsung).

Quality criteria ensuring accurate mPTS measurement on lateral radiographs were based on previous reports and are as follows: tibial length of ≥12.5 cm, ≤5 mm rotation measured at the posterior femoral condyles and ≤5 mm abduction/adduction measured at the distal femur condyles. Measurements of the mPTS on pre‐ and post‐operative radiographs followed the method described by Dejour and Bonnin [9], using the medial tibial plateau as a reference point in native knee joints and the tibial implant as a reference point in knee joints after UKA. To determine the diaphyseal tibial axis, a line was drawn between two points at equal distances from the anterior and posterior borders of the tibia—one just below the tibial tubercle and the other 10 cm distally. A reference line perpendicular to this axis was then drawn at the level of the tibiofemoral joint. The posterior inclination of the tibial plateau was measured by drawing a line connecting the most superior points of the anterior and posterior edges of the medial tibial plateau (or the tibial component post‐operatively). The angle between this inclination line and the perpendicular reference line was defined as the mPTS.

**Figure 1:**
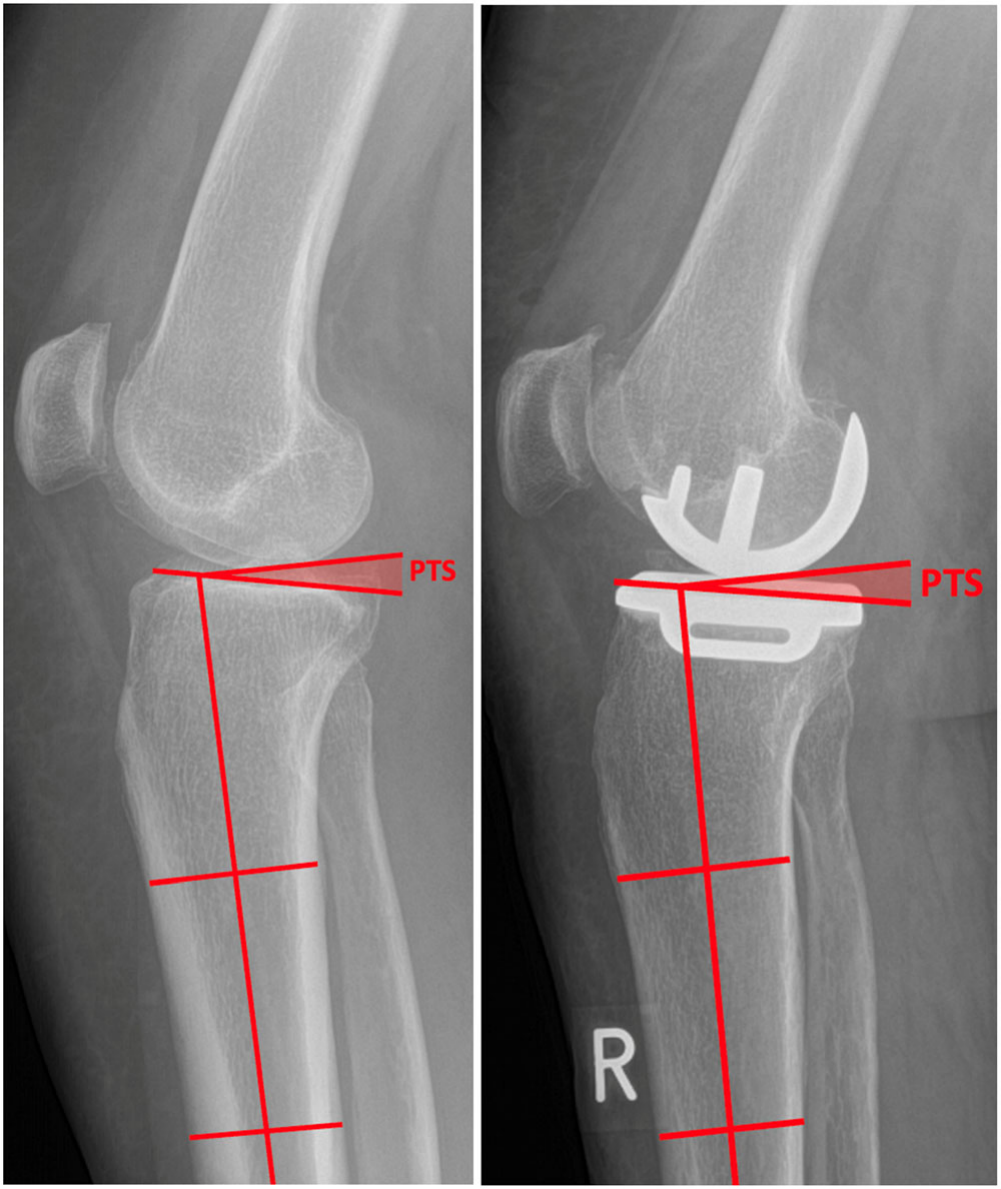
Measurements of mPTS. Left: mPTS lateral preoperative knee radiograph showcasing mPTS measurement. Right: mPTS lateral post‐operative knee radiograph showcasing mPTS measurement. mPTS, medial posterior tibial slope.

**Figure 2:**
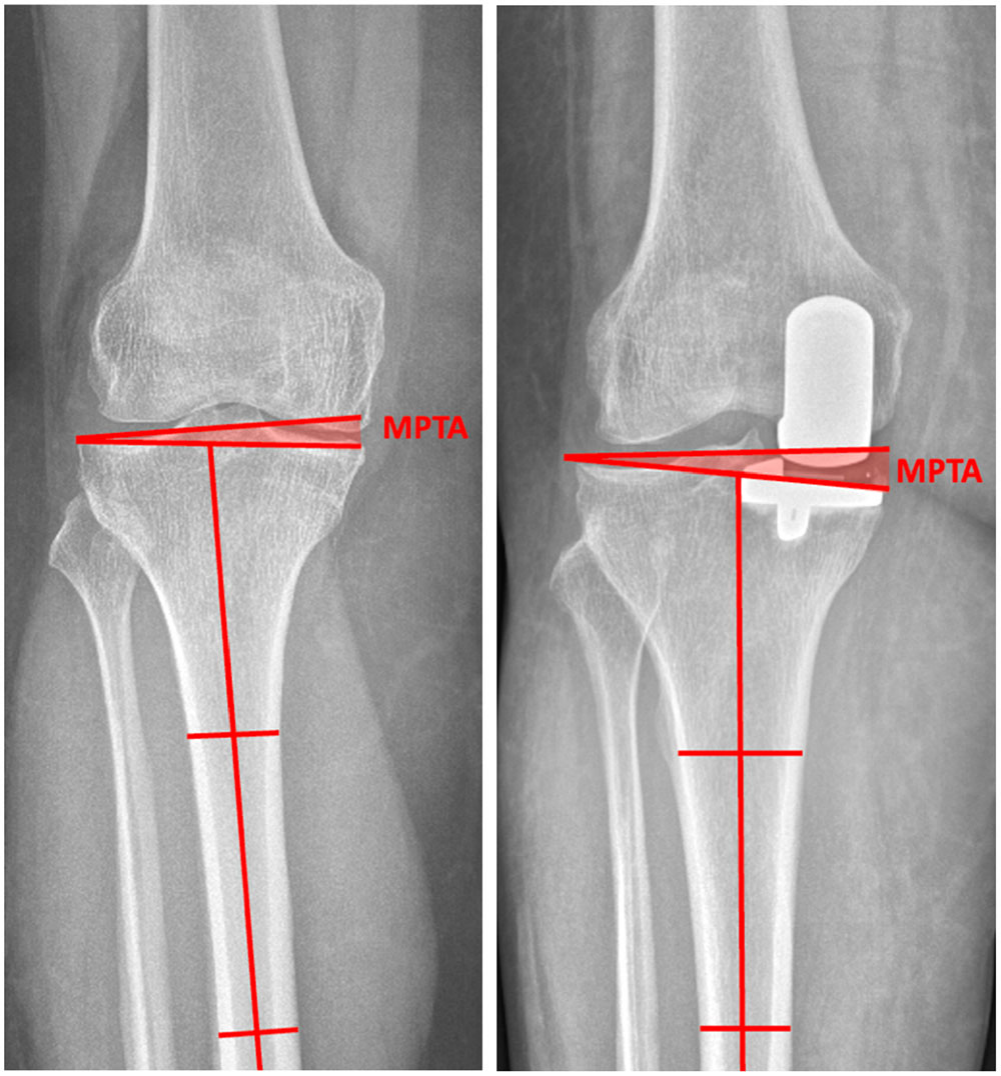
Measurements of MPTA. Left: MPTA anterior to posterior preoperative knee radiograph showcasing MPTA measurement. Right: MPTA anterior to posterior post‐operative knee radiograph showcasing MPTA measurements. MPTA, medial proximal tibial angle.

MPTA was measured on pre‐ and post‐operative radiographs following the method described by Petersen and Engh [29, 40]. The tibial anatomic axis was determined by identifying two points at the centre of the tibial cortex—one located 10 cm distal to the joint line and the other at the most distal point visible on the radiograph. A perpendicular line to this axis was drawn at the level of the tibiofemoral joint. Another line was drawn along the tibial plateau, extending from the lateral edge to the medial edge, or to the most superior medial edge of the tibial component on post‐operative images. The angle between this line and the perpendicular reference line was defined as the MPTA. Measurement techniques are presented in Figures 1 and 2.

Two independent observers, blinded to patient clinical data, performed measurements of mPTS and MPTA. Intraclass correlation coefficients for interobserver reliability were 0.85 (preoperative) and 0.94 (post‐operative) for mPTS and 0.94 (preoperative) and 0.99 (post‐operative) for MPTA, indicating excellent agreement [19].

Measurements were carried out using a PACS workstation (Centricity RIS‐I 4.2 Plus, GE Healthcare).

### Statistics

A power analysis (Cohen's *d* = 0.5, *a* = 0.05, power = 80%) was conducted to determine the required sample size. A minimum of 88 patients was required to detect a significant difference, confirming the adequacy of our study population (*n* = 96).

Descriptive parameters were analyzed, and mean, standard deviation and range were calculated wherever applicable. For quantitative variables, the normality of distribution was assessed using the Shapiro–Wilk normality test. A two‐sample *t* test was conducted when means were normally distributed, otherwise the Wilcoxon signed‐rank test for dependent samples was used to test for significance. One‐sample *t* test with a hypothesized population mean of 7.00 for the target post‐operative mPTS was performed to test for the statistical difference of post‐operative mPTS from planned mPTS. Statistical significance was set at *p* < 0.05.

Additionally, cases were analyzed according to the following subgroups: cases within ±1°, ±2° and ±3° of the planned mPTS (7°) to post‐operative mPTS, and cases with <3° or ≥3° of pre‐ to post‐operative change in mPTS. Distribution of cases according to groups, pre‐ and post‐operative mPTS and MPTA, and pre‐ to post‐operative changes in mPTS and MPTA were reported and analyzed for statistical significance. Analysis of variance (ANOVA) was applied to test for significant differences in the reported MPTA change between groups.

To report on the predictive quality of mPTS change on MPTA change, the Pearson correlation coefficient was calculated. A Pearson correlation coefficient (*r*) of 0–0.19 was regarded as very weak, 0.2–0.39 as weak, 0.40–0.59 as moderate, 0.6–0.79 as strong and 0.8–1 as very strong correlation [33].

All calculations were performed using Microsoft Excel for Microsoft (Version 16, 17628.20006, Microsoft) and SPSS (IBM Corp. Released 2023. IBM SPSS Statistics for Windows, Version 29.0.2.0).

### Ethical aspects

The study protocol was approved by the ethics committee of Charité – Universitätsmedizin Berlin (EA2/016/21) and was conducted in accordance with the Declaration of Helsinki. Written informed consent was obtained from all patients.

## RESULTS

A total of 96 consecutive patients (51 [53.13%] females and 45 [46.87%] males) met the inclusion criteria.

The mean preoperative mPTS was found at 9.27° (SD, 3.41°; range, 0.39–17.78°), and the mean post‐operative mPTS at 7.25° (SD, 2.23°; range, 2.27–12.94°), with a statistically significant average pre‐ to post‐operative mPTS change of −2.02° (SD, 3.84°; range, −10.84° to 6.63°; *p* < 0.001). Mean differences from the targeted mPTS of 7°, as well as number of cases within ±1°, ±2° and ±3° of 7°, are presented in Table 2.

**Table 2:** mPTS change and accuracy.

	Preoperative mPTS	Post‐operative mPTS	Pre‐ to post‐operative mPTS change	Cases within ±1° of 7°	Cases within ±2° of 7°	Cases within ±3° of 7°	Mean difference from 7°	*p* **value**
*n* = 96 (100%)	9.27° (SD, 3.41°)	7.24° (SD, 2.23°)	−2.03° (SD, 3.7°)	35 (38.04%)	66 (71.74%)	78 (84.78%)	−0.25° (SD, 2.26°)	0.142

*Note*: Values presented represent means if not stated otherwise.

Abbreviations: mPTS, medial posterior tibial slope; SD, standard deviation.

Pre‐ and post‐operative MPTA values were 85.39° (SD, 2.34°; range, 80.29–92.35°) and 84.12° (SD, 2.55°; range, 79.22–98.57°), respectively, with an average pre‐ to post‐operative MPTA change of −1.28° (SD, 1.93°; range, −5.49° to 6.22°; *p* < 0.001).

A post‐operative mPTS within ±2° of targeted mPTS was achieved in 71.7% of patients. The changes in MPTA and mPTS across groups are detailed in Table 3.

**Table 3:** Pre‐ and post‐operative mPTS and MPTA according to groups.

	Preoperative	Post‐operative	Difference	*p* **value**
All cases *n* = 96 (100%)				
mPTS	9.27° (SD, 3.41°)	7.24° (SD, 2.23°)	−2.03° (SD, 3.7°)	<0.001
MPTA	85.39 (SD, 2.34°)	84.12° (SD, 2.55°)	−1.28° (SD, 1.93°)	<0.001
Cases within ±1° of 7° *n* = 35 (38.04%)				
mPTS	9.25° (SD, 3.59°)	7.05° (SD, 0.49°)	−2.20° (SD, 3.44°)	<0.001
MPTA	85.42° (SD, 2.49°)	83.96° (SD, 2.39°)	−1.46° (SD, 1.70°)	<0.001
Cases within ±2° of 7° *n* = 66 (71.74%)				
mPTS	9.41 (SD, 3.50°)	6.98° (SD, 1.11°)	−2.42° (SD, 3.35°)	<0.001
MPTA	85.30° (SD, 2.56°)	83.94° (SD, 2.84°)	−1.36° (SD, 2.00°)	<0.001
Cases within ±3° of 7° *n* = 78 (84.78%)				
mPTS	9.31° (SD, 3.47°)	6.99° (SD, 1.43°)	−2.33° (SD, 3.59°)	<0.001
MPTA	85.28° (SD, 2.48°)	84.00° (SD, 2.72°)	−1.28° (SD, 1.88°)	<0.001
Cases with <3° mPTS change *n* = 48 (50%)				
mPTS	8.13° (SD, 2.42°)	7.59° (SD, 2.24°)	−0.54° (SD, 1.71°)	0.04
MPTA	85.73° (SD, 2.07°)	84.41° (SD, 2.65°)	−1.32° (SD, 2.02°)	<0.001
Cases with ≥3° mPTS change *n* = 48 (50%)				
mPTS	10.42° (SD, 3.86°)	6.90° (SD, 2.18°)	−3.52° (SD, 4.72°)	<0.001
MPTA	85.06° (SD, 2.57°)	83.82° (SD, 2.43°)	−1.24° (SD, 1.85°)	<0.001

*Note*: Values presented represent means if not stated otherwise.

Abbreviations: MPTA, medial proximal tibial angle; mPTS, medial posterior tibial slope; SD, standard deviation.

As displayed in Table 4, the Pearson correlation coefficients between mPTS and MPTA change in all cases, as well as according to groups, were very weak. ANOVA analysis revealed no statistically significant difference in MPTA change across groups (*H* = 0.218; df = 4; *p* = 0.994). The target range for the MPTA was met by the majority of all cases (80.21%) and regardless of the amount of mPTS change (in cases with ≥3 mPTS change: 70.84%).

**Table 4:** Correlation analysis.

Pre‐ to post‐operative mPTS change to pre‐ to post‐operative MPTA change	Pearson correlation coefficient (*r*)	*p* **value**
All cases *n* = 96 (100%)	−0.0492	0.634
Cases within ±1° of 7° *n* = 35 (38.04%)	−0.112	0.523
Cases within ±2° of 7° *n* = 66 (71.74%)	−0.059	0.633
Cases within ±3° of 7° *n* = 78 (84.78%)	−0.037	0.748
Cases with <3° mPTS change *n* = 48 (50%)	0.136	0.357
Cases with ≥3° mPTS change *n* = 48 (50%)	−0.125	0.398

Abbreviations: MPTA, medial proximal tibial angle; mPTS, medial posterior tibial slope.

## DISCUSSION

The most important finding of this study was that a post‐operative mPTS within ±2° of the targeted PTS can be achieved in most patients undergoing UKA (71.7%), confirming our first hypothesis. Additionally, no significant correlation was found between changes in mPTS and MPTA, and the majority of cases (70.84%) with an mPTS change of ≥3° had an MPTA within the targeted range, thereby confirming our second hypothesis.

While in most patients, the post‐operative mPTS did not differ by more than 2° from the targeted mPTS of 7°, the importance of this precise adjustment [or an even more accurate modification through robotics [37]] remains unclear. In a finite element model of a mobile‐bearing medial UKA, an increased mPTS of 10° led to elevated anterior translation and external rotation of the tibia during flexion, as well as a ventral shift of the mobile‐bearing inlay [39]. Conversely, in native knees, an increase in anterior tibial translation due to an elevated mPTS was observed only in a static position, but not dynamically. This suggests the presence of a dynamic compensatory mechanism absent in either the finite element model and/or UKA [42]. Chen et al. [8] reported no significant difference in functional outcomes between patients with different post‐operative mPTS values, while Pourzal et al. [31] found that excessive tibial slope changes increased polyethylene wear rates. These findings align with our study's results, highlighting the need for further longitudinal research.

However, there is stronger evidence regarding the importance of accurately reconstructing the patient's MPTA. Maintaining an optimal MPTA is crucial to prevent excessive varus or valgus alignment, which may accelerate polyethylene wear and implant loosening [7, 17, 22]. Hernigou and Deschamps [13] reported that valgus overcorrection in UKA increases lateral compartment degeneration risk, reinforcing the importance of precise intraoperative tibial resections.

Consequently, most of the current evidence suggests a slight under‐correction of the MPTA with persistent residual varus [1, 27]. However, these suggestions raise the question of what should be considered a physiological MPTA. While the manufacturer of the knee system used in this study sets the target value at 90°, recent studies on anatomical MPTA have revealed an average MPTA of 86.6° in European populations [26].

Regardless of the target values, precise surgical control over bony alignment parameters is essential. In native knees, medial opening wedge high tibial osteotomy can significantly alter the MPTA without affecting the individual PTS [24]. Conversely, this study demonstrated that targeted modification of the mPTS is achievable in conventional UKA without impacting the MPTA, as no correlation was found between changes in mPTS and MPTA. The target range for MPTA was met in the majority of cases, even when a significant mPTS change occurred.

In addition to the limitations inherent to the retrospective design of this study, there are others worth mentioning. First, this study reports on UKA performed by a single, highly experienced surgeon at a single institution. While this ensures consistency in surgical technique and decision‐making, it also introduces potential bias, as outcomes may not be generalizable to surgeons with different skill levels. Additionally, we investigated only cases using the widely used Oxford UKA system, so the conclusions drawn may not apply to other systems with different instrumentation. Finally, while this study focuses on radiographic alignment changes, it does not assess functional outcomes such as pain relief or knee function improvement. Future studies should integrate patient‐reported outcome measures (e.g., Oxford Knee Score, Western Ontario and McMaster Universities Osteoarthritis Index) to correlate alignment modifications with clinical benefit.

## CONCLUSION

Targeted modification of the mPTS is achievable in the majority of patients undergoing manual, non‐robotic‐assisted UKA. Changes in MPTA during implantation do not correlate with changes in mPTS and remain within the target range even in patients with severe surgical mPTS alteration.

## AUTHOR CONTRIBUTIONS

Each named author has substantially contributed to conducting the underlying research and drafting this manuscript.

## CONFLICT OF INTEREST STATEMENT

The authors declare no conflicts of interest.

## ETHICS STATEMENT

The study protocol was approved by the local ethics committee (EA2/016/21), and the study was conducted in accordance with the Declaration of Helsinki. Written informed consent was obtained from all patients included in this study.

## Data Availability

The data that support the findings of this study are available from the corresponding author upon reasonable request.
